# Anatomy of Adipose Compartments and Fascial Structures in the Posterolateral Region of the Kidney With Special Focus on the Thin Adipose Compartment

**DOI:** 10.1111/iju.70303

**Published:** 2025-11-27

**Authors:** Atsuhiko Ochi, Satoru Muro, Sho Mitsumaru, Akimoto Nimura, Keiichi Akita

**Affiliations:** ^1^ Department of Urology Kameda Medical Center Chiba Japan; ^2^ Department of Clinical Anatomy Institute of Science, Tokyo Tokyo Japan; ^3^ Department of Functional Joint Anatomy, Biomedical Engineering Laboratory Institute of Industry Incubation, Institute of Science, Tokyo Tokyo Japan

**Keywords:** laparoscopic surgery, lateroconal fascia, pararenal fat, renal fascia, retroperitoneal space

## Abstract

**Objectives:**

To anatomically and histologically define the adipose and fascial structures posterior and lateral to the kidney and propose a compartment‐based anatomical model aligned with intraoperative observations.

**Methods:**

Seven cadavers were used for macroscopic and histological analyses. In the macroscopic analysis, the spatial relationships between the perirenal fat (PeRF), pararenal fat, posterior renal fascia (PRF), and extraperitoneal fascia (EPF) were examined. Histological observations focused on the distribution and continuity of adipose compartments and the organization of the surrounding dense fibrous connective tissue.

**Results:**

Macroscopically, the EPF covered the anteromedial pararenal fat and extended posteriorly to the kidney. Upon incision, a small amount of adipose tissue was observed directly beneath it. Removing this TAC exposes the peritoneum and PRF with a clear demarcation between them. Histological analysis confirmed that the posterior renal and EPF were distinct, with dense connective tissue structures enclosing a separate TAC. This compartment extended anteriorly between the PeRF and peritoneum, and laterally between the peritoneum and EPF. These extensions converge near the peritoneal reflection in the lateral renal region, forming a characteristic triradiate configuration of adipose tissue.

**Conclusions:**

Our findings challenge the classical notion that the renal fascia is a single continuous layer, supporting a compartment‐centered anatomical model. The posterior and lateral regions of the kidney contain a distinct third adipose compartment, bordered by the posterior renal and extraperitoneal fasciae. This model offers improved anatomical clarity and may aid understanding during laparoscopic, retroperitoneoscopic, and robot‐assisted surgeries.

**Abbreviations**
AC: ascending colon
ARF: anterior renal fascia
AW: abdominal wall
AWM: abdominal wall musculature
CT: computed tomography
EPF: extraperitoneal fascia
GB: gallbladder
IP: interfascial plane
K: kidney
LCF: lateroconal fascia
PaRF: pararenal fat
PeRF: perirenal fat
PM: psoas muscle
Pn: peritoneum
PRF: posterior renal fascia
QL: quadratus lumborum muscle
RF: renal fascia
TAC: thin adipose compartment
TC: transverse colon
TF: transversalis fascia

## 1 Introduction

The renal fascia (RF) is a fibrous membranous structure located in the retroperitoneal region that envelops the kidneys, adrenal glands, ureters, gonadal vessels, and perirenal fat (PeRF) from anterior and posterior aspects. Zuckerkandl first described the posterior RF (PRF), followed by Gerota identifying the anterior RF (ARF) [1, 2, 3]. These fasciae converge laterally to form the lateroconal fascia (LCF) [4]. Collectively, they function as mechanical barriers that protect retroperitoneal organs from external forces and the spread of inflammation or infection, and as critical anatomical landmarks that influence radiological interpretation, intraoperative visualization, and dissection plane selection during surgical procedures.

Traditionally, the fascia of the retroperitoneal region is regarded as a single, continuous, membranous structure [5], as reflected in current anatomical textbooks [6]. However, since the advent of computed tomography (CT) in the 1980s, radiologists have proposed the concept of the interfascial plane (IP), suggesting that the ARF, PRF, and LCF each consist of multilayered fibrous membranes [7, 8]. These layers contain potential spaces that may serve as pathways for fluid components such as inflammatory exudates. Based on CT imaging and cadaveric studies, Marks et al. reported that the PRF could be divided into two distinct membranous structures: one connected to the LCF and the other connected to the ARF [9]. During retroperitoneal surgery, the outermost membranous structure within the pararenal fat (PaRF; flank pad) on the posterior aspect of the kidney is sometimes referred to as the LCF to distinguish it from the PRF [10, 11, 12], although this usage deviates from the original anatomical definition. These observations challenge the conventional model of a single PRF, suggesting its insufficiency in fully representing the complex tissue organization in the posterior and lateral regions of the kidney.

This study aimed to anatomically and histologically investigate the configuration of adipose tissue compartments and surrounding dense connective tissue structures in the posterior and lateral regions of the kidney from a perspective distinct from the conventional RF theory. Ochi et al. [13] previously reexamined the internal architecture of the RF through a novel framework of adipose tissue compartmentalization (zoning). In this study, we applied this concept to investigate the distribution and spatial relationships of adipose tissue in the retroperitoneal region, lateral and posterior to the kidney, and interpreted the dense fibrous connective tissue membranes that delineate fat compartments as fasciae. This fat‐based approach may provide a more accurate anatomical model of the retroperitoneum and enhance the interpretation of findings during laparoscopic, retroperitoneoscopic, and robot‐assisted surgeries.

## 2 Methods

### 2.1 Preparation of Cadaveric Specimens

Seven adult male cadavers (mean age at death, 81.7 [range, 73–92] years) were donated to our department in accordance with Japanese law entitled The Act on Body Donation for Medical and Dental Education (Act No. 56 of 1983). All donors voluntarily agreed that their remains would be used for educational and research purposes before their death. Written informed consent was obtained from the bereaved families, and no objections were raised. All cadavers were fixed in 8% formalin via arterial perfusion and preserved in 30% alcohol to maintain tissue integrity and prevent fungal growth. Cadavers with histories of major abdominal or pelvic surgery were excluded.

### 2.2 Macroscopic Anatomy

Three of the seven cadavers were used for macroscopic analysis. Following sample retrieval, two specimens were used for macroscopic cross‐sectional observations, and the remaining specimens were used for layer‐by‐layer anatomical dissection. For macroscopic observation, each specimen was sectioned horizontally using a diamond band saw to obtain cross‐sectional images of the kidneys. During the anatomical dissection of the layered structures, a midline laparotomy was performed, and the right abdominal wall (AW) was laterally reflected. Subsequently, the peritoneum and extraperitoneal fascia (EPF) were carefully separated en bloc from the underlying adipose tissue and reflected medially, thereby exposing the fascial structures that enclosed the kidney and PeRF.

### 2.3 Histological Analysis

Histological analyses were performed on the remaining four cadavers. A single transverse section at the level intersecting the kidney was prepared using a diamond band saw (EXAKT 312; EXAKT Technologies Inc., Germany) following a previously described methodology [14, 15]. Representative tissue blocks containing the lateral and posterior regions of the kidneys were excised for histological examination. All tissue blocks were fixed in 10% neutral‐buffered formalin for 24 h, dehydrated using a graded ethanol series (70%, 80%, 90%, and 100%), cleared in xylene, and embedded in paraffin under negative pressure. The paraffin was replaced three times over 5 days to ensure complete infiltration. Paraffin sections (5‐μm thick) were prepared using a rotary microtome (RM2235; Leica Biosystems Nussloch GmbH, Wetzlar, Germany) and stained with Elastica van Gieson staining. In the present observations, we defined the ARF as the portion of the RF located anterior to the peritoneal reflection and the PRF as the portion located posterior to the peritoneal reflection.

## 3 Results

### 3.1 Macroscopic Findings

**FIGURE 1:**
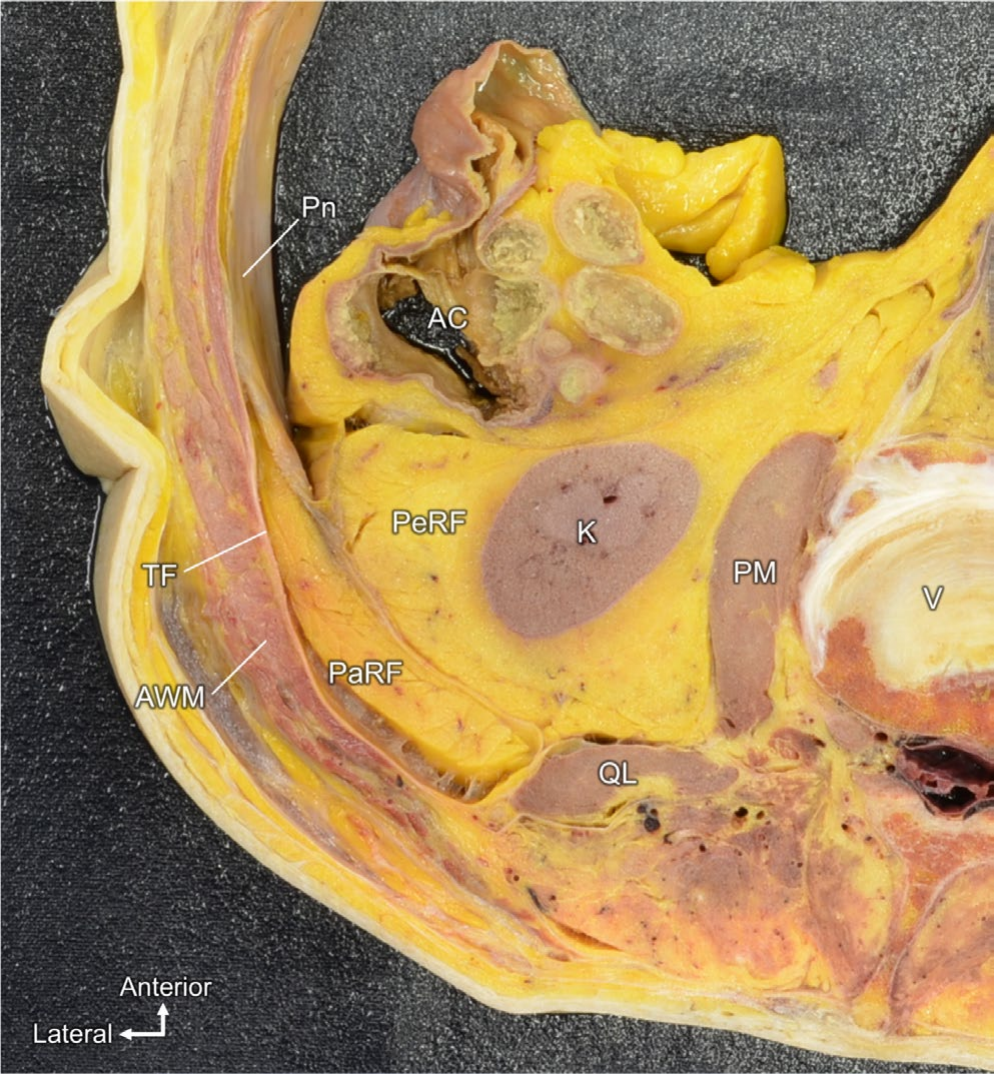
Peritoneal reflection and fat compartments around the kidney. Transverse abdominal sections through the kidneys. The peritoneum covers the posterior abdominal wall, including the ascending colon and mesocolonic fat, and reflects the lateral abdominal wall. The pararenal fat (PaRF) lies anteromedial to the abdominal wall muscles, whereas the perirenal fat (PeRF) surrounds the kidney. The boundary between the PaRF and PeRF is distinct, with a peritoneal reflection positioned between them.

The peritoneum lining the abdominal cavity was identified in a transverse abdominal section through the kidneys (Figure 1). The peritoneum covered the posterior AW, including the ascending colon and its mesocolonic fat, and was then reflected laterally along the inner surface of the lateral AW. The abdominal wall musculature (AWM) extended from the lateral wall toward the posterior side, anteromedial to these muscles, and the fat compartment corresponding to the PaRF was observed. PeRF surrounding the kidney was visible medially and anteriorly to the PaRF. The boundary between the PaRF and PeRF was clearly demarcated in the cross‐section. The peritoneal reflection was located between PaRF and PeRF.

**FIGURE 2:**
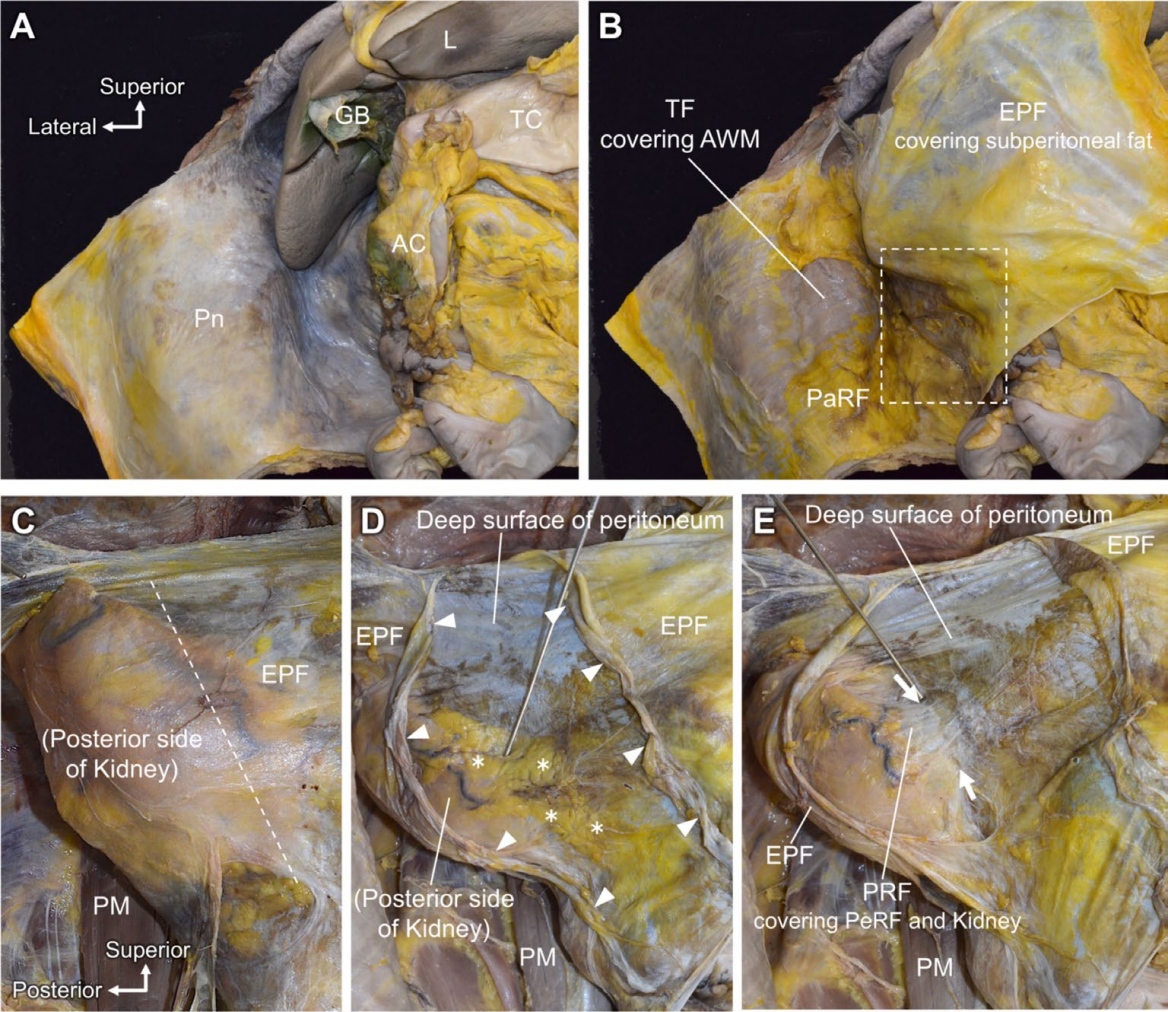
Macroscopic anatomy of the posterior and lateral retroperitoneal region. (A) Anterior aspect of the right half of the abdomen. The peritoneum lining the right abdominal wall continues from the anterior to the lateral and posterior abdominal walls. (B) After the peritoneum and EPF are dissected and reflected medially, PaRF are observed on the TF covering AWM. (C) Enlarged view of the dashed square in B. The EPF extends posteriorly to cover the posterior aspect of the perirenal region. (D) The EPF is cut along the dashed line in C. Following longitudinal incision and reflection of the EPF, a TAC (asterisk) is observed immediately beneath the EPF. The arrowhead indicates the edge of the EPF incision. (E) After removal of the adipose layer, PRF enclosing PeRF is clearly exposed. The arrow indicates the border between the peritoneum and the PRF. AC, ascending colon; AWM, abdominal wall musculature; EPF, extraperitoneal fascia; GB, gallbladder; L, liver; PaRF, pararenal fat; PeRF, perirenal fat; PM, psoas muscle; Pn, peritoneum; PRF, posterior renal fascia; TC, transverse colon; TF, transversalis fascia.

The peritoneum lining the right AW extended seamlessly from the anterior to the lateral and posterior AWs (Figure 2A). When the peritoneum and EPF were dissected together from the AW and reflected medially, the transversalis fascia (TF) covering the muscular layer of the AW and adipose tissue attached to its anterior surface became clearly visible (Figure 2B). The adipose tissue extending from the posterior aspect of the kidney to the AW was identified as the PaRF. A thin layer of adipose tissue was observed between the peritoneum and EPF (Figure 2B). The EPF continued posteriorly and covered the dorsal aspects of the perirenal structures (Figure 2C). Upon longitudinal incision and careful bilateral reflection of the EPF, a thin layer of adipose tissue was observed immediately beneath it (Figure 2D, asterisk). After removing the adipose layer, the deep surface of the peritoneum was exposed anteriorly. Subsequently, a membranous structure enclosing the kidney and PeRF was exposed and identified as the PRF (Figure 2E). The PRF extended anteriorly and formed a boundary with the peritoneum at the point where it passed in front of the PeRF.

### 3.2 Histological Findings

**FIGURE 3:**
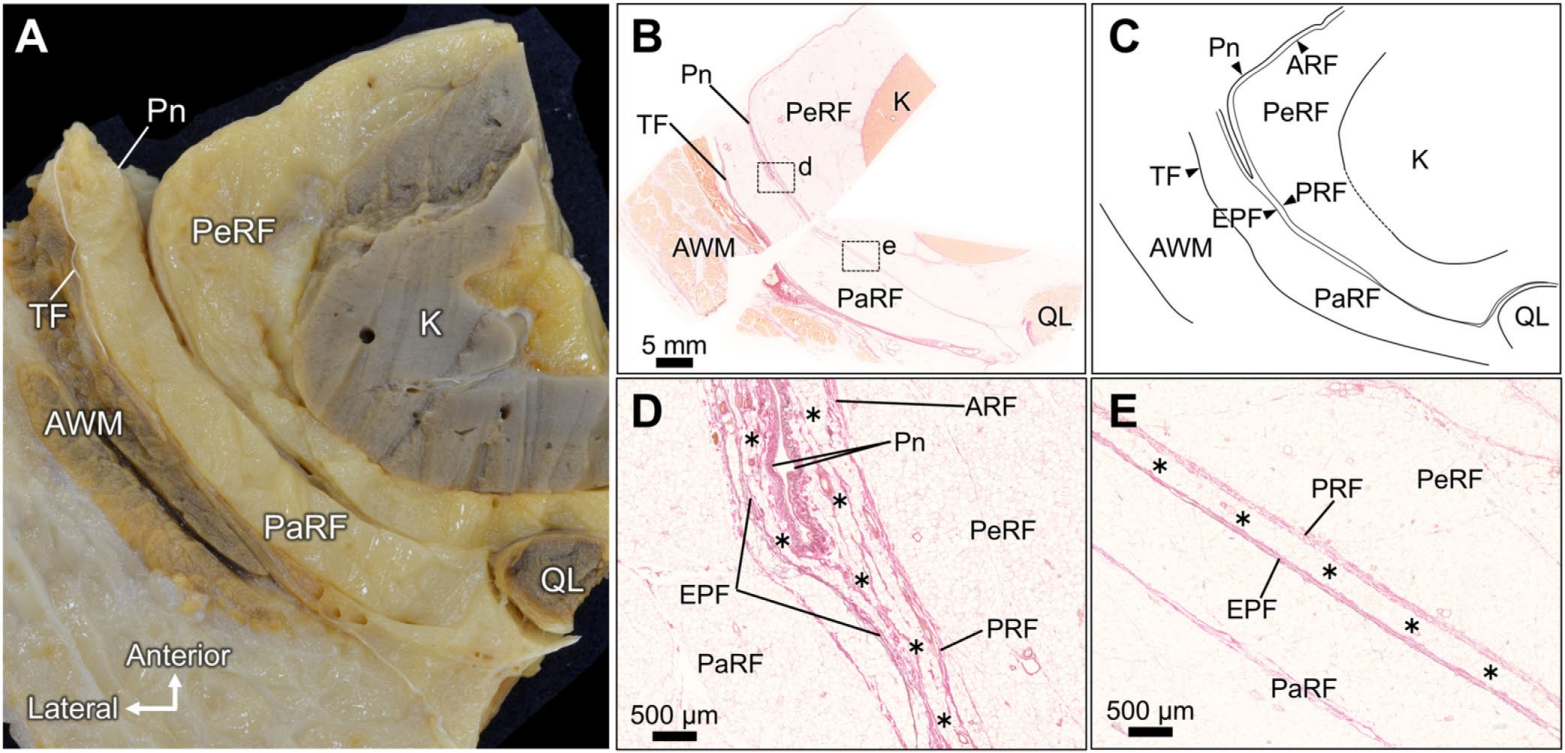
Histological examination of the posterior and lateral adipose compartments and fasciae. (A) Cross‐sectional overview showing PeRF surrounding the kidney, PaRF located posterolaterally, and the abdominal wall muscles with the TF.(B) Low‐magnification histological image showing the spatial distribution of the PeRF and PaRF, the peritoneal reflection, and surrounding fascial structures. (C) Schematic illustration corresponding to panel B.(D, E) D is an enlarged view of the dashed square d in Figure 3B,E is an enlarged view of the dashed square in Figure 3B. Higher‐magnification images demonstrate a TAC (asterisks) interposed between the peritoneum and EPF laterally, between the peritoneum and ARF anteriorly, and between the PRF and EPF posteriorly. These three extensions converge at the peritoneal reflection, forming a triradiate adipose tissue configuration. In the posterior region of the kidney, this TAC is bordered by dense connective tissue structures: EPF posterolaterally and PRF anteromedially.AWM, abdominal wall musculature; EPF, extraperitoneal fascia; PaRF, pararenal fat; PeRF, perirenal fat; Pn, peritoneum; PRF, posterior renal fascia; QL, quadratus lumborum muscle; TF, transversalis fascia.

In cross‐sectional observations of the retroperitoneal region, the PeRF was located adjacent to the kidney, whereas a distinct adipose compartment identified as the PaRF was found posterolateral to the PeRF (Figure 3A). The PeRF was distributed around the anterior, lateral, and posterior aspects of the kidney and restricted to the perirenal region without extending into adjacent extrarenal areas. Conversely, the PaRF was located posterior and lateral to the PeRF and extended anteriorly toward the lateral AW. Lateral to the PaRF, the AWM was observed.

Histological examination clearly demonstrated the adipose compartments of PeRF and PaRF, including peritoneal reflections and dense connective tissue structures enclosing these fat compartments (Figure 3B,C). Higher magnification views revealed a thin adipose compartment (TAC) (asterisks in Figure 3D) interposed between the peritoneum and ARF and between the peritoneum and EPF. This TAC was located between the peritoneum and PaRF in the lateral AW and between the peritoneum and PeRF in the posterior AW. These TACs converge at the inflection point of the peritoneal reflection, forming a triradiate configuration of fat compartments. From this junction, the tissue extended posteriorly into the dorsal region to the PeRF (Figure 3D,E). The TAC became progressively thinner toward the medial posterior side of the kidney, and eventually lost its adipose content, at which point the EPF and PRF merged. However, the exact site of convergence was inconsistent and varied among the examined cadavers. Distinct dense connective tissue membranes demarcated the TAC from the PeRF and PaRF. The former was identified as the PRF and the latter as the EPF. The TACs at each site and their triradiate convergence were consistently observed in all four cadavers examined.

## 4 Discussion

**FIGURE 4:**
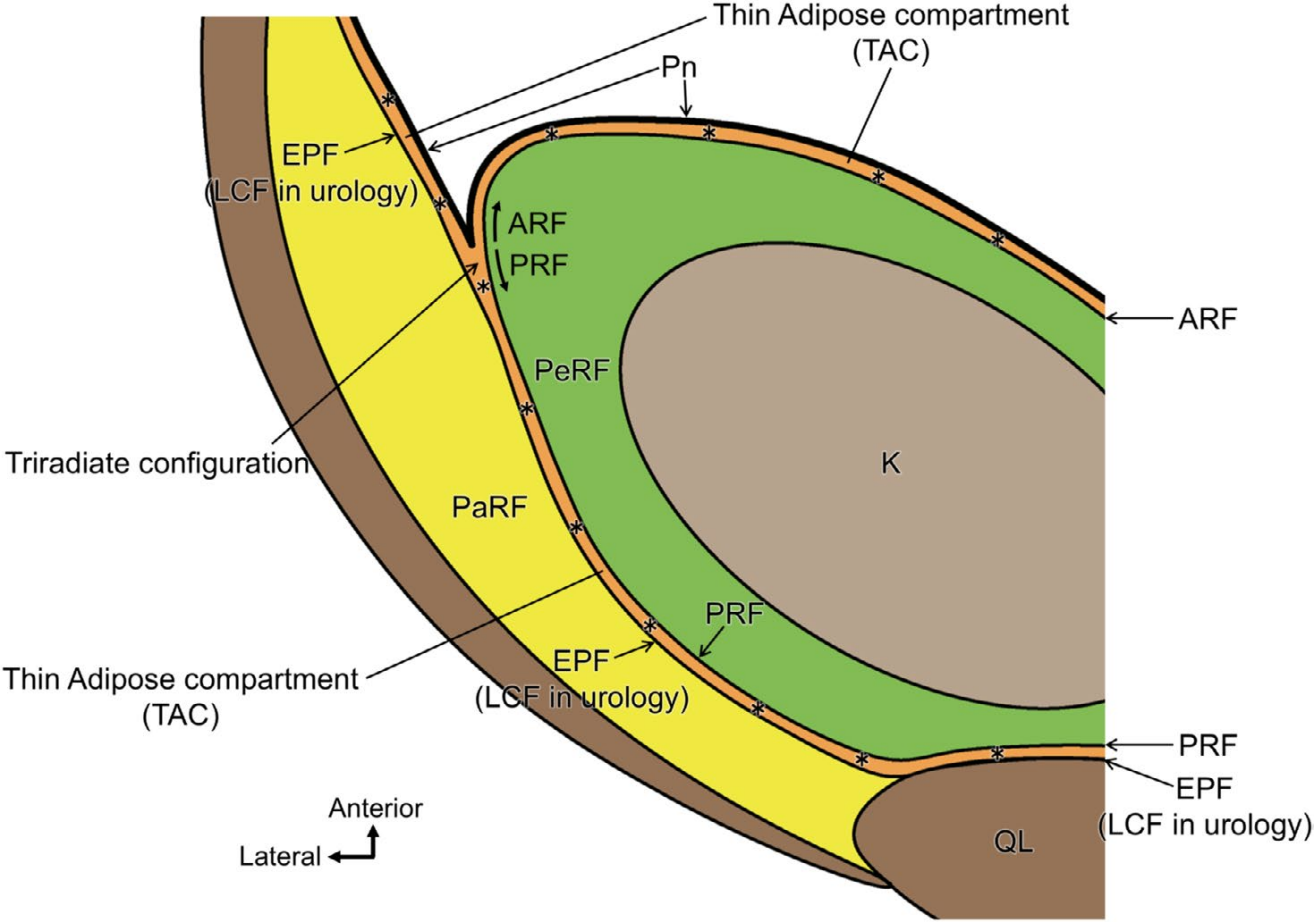
Schematic representation of the adipose tissue compartments and surrounding fascial structures in the posterolateral retroperitoneal region. This diagram illustrates the spatial configuration of the three distinct adipose tissue compartments (asterisks): Perirenal fat (PeRF), pararenal fat (PaRF), and an interposed TAC. The TAC is bordered anteromedially by the posterior renal fascia (PRF) and posterolaterally by the extraperitoneal fascia (EPF), both of which are dense fibrous connective tissue layers. This TAC continues anteromedially between the ARF and peritoneum and anterolaterally between the EPF and peritoneum. These three extensions converged at the lateral margin of the kidney, forming a triradiate configuration. This arrangement challenges the classical unilaminar concept of the Gerota fascia and supports a compartment‐based anatomical model.

This investigation clarified the structure of adipose tissue compartments located posterior and lateral to the kidney and the arrangement of the surrounding membranous structures. Two principal findings were obtained (Figure 4). First, the EPF was observed as an independent membranous structure along the posterior region of the kidney, clearly distinct from the PRF. Second, a TAC interposed between the PaRF and PeRF was continuous with the TACs situated between the peritoneum and PaRF and between the peritoneum and PeRF. These three extensions converge in the lateral region of the kidney to form a triradiate configuration of adipose tissue. These findings indicate that the classical single‐layer model of the renal and LCF is insufficient to account for the actual anatomical complexity of the retroperitoneal region, necessitating a reevaluation of the current structural interpretations.

The PRF was first described by Zuckerkandl in 1883 as a fibrous membrane along the dorsal aspect of the kidneys. Subsequently, Gerota identified an analogous fibrous structure on the anterior side of the kidney called the ARF [3]; both layers are now commonly referred to collectively as the Gerota fascia [2, 3, 16, 17]. Tobin [5] further defined the retroperitoneum as the region between the parietal peritoneum and TF. According to this framework, the RF is a condensation of the retroperitoneal connective tissue that envelops the kidneys, surrounding fat, adrenal glands, gonadal vessels, and major vasculature. Gerota and Tobin believed that the ARF and PRF fuse laterally in a region referred to as the subperitoneal fascia. This structure was later termed the LCF based on anatomical dissections [4]. With the advent of CT, the PRF was recognized as thicker than its anterior counterpart. Marks et al. [9] demonstrated that the PRF could be divided into two distinct layers: one continuous with the ARF and the other with the LCF. These findings laid the foundation for the concept of the “IP,” wherein the fascial structures—anterior, posterior, and LCF—are viewed as multilayered membranes containing potential spaces that serve as conduits for fluid, inflammation, or hemorrhage [7, 8, 18]. In modern urologic surgery, particularly with retroperitoneal approaches, the fibrous membrane covering the medial surface of the PaRF and the lateral border of the PeRF is often identified as the LCF and distinguished from the RF [10, 11, 12].

All previous models are fascia‐centered. Our study proposes an adipose compartment model that places fat organization, rather than fasciae, at the core of anatomical delineation, providing a more accurate intraoperative representation. Table 1 summarizes the correspondence between the conventional RF and IP models and our adipose compartment framework. Recognition of TAC in the posterior, lateral, and anterior regions allows interpretation of surrounding dense connective tissues (PRF, EPF, and ARF) as continuous structures. The absence of TAC recognition in earlier models caused inconsistencies in fascial interpretation. Owing to its thinness (~500 μm), TAC is not clearly visible macroscopically or on standard CT; its continuity was demonstrated histologically. Future high‐resolution or photon‐counting CT—and certain pathological fluid collections—may enable its indirect or direct visualization.

**TABLE 1:** Comparison of anatomical interpretations of the perirenal region.

Tissue composition and distribution	Posterior region of the kidney			Lateral region of the kidney			Anterior region of the kidney		
	Dense connective tissue covering the PeRF	TAC	Dense connective tissue covering the PaRF	Basal lamina of the peritoneum	TAC	Dense connective tissue covering the PaRF	Basal lamina of the peritoneum	TAC	Dense connective tissue covering the PeRF
Renal fascia model (Anatomy: Standring 2020 [6]; MacLennan 2012 [17])	PRF	? (Unrecognized)	PRF	Peritoneum	Subperitoneal fat	LCF	Peritoneum	Subperitoneal fat	ARF
Renal fascia model (Urology: Yin et al. 2016 [10]; Alimu et al. 2022 [11]; Takahashi 2012 [12]; Huang et al. 2018 [18])	PRF	? (Unrecognized)	LCF	Peritoneum	Subperitoneal fat	LCF	Peritoneum	Subperitoneal fat	ARF
Interfascial plane model (Radiology: Molmenti et al. 1996 [7]; Aizenstein et al. 1997 [8])	PRF (Plane)			LCF (Plane)			ARF (Plane)		
Adipose compartment model (The present study)	PRF	Posterior TAC	EPF	Peritoneum	Lateral TAC	EPF	Peritoneum	Anterior TAC	ARF

Abbreviations: ARF, anterior renal fascia; EPF, extraperitoneal fascia; LCF, lateroconal fascia; PaRF, pararenal fat; PeRF, perirenal fat; PRF, posterior renal fascia; TAC, thin adipose compartment.

The presence of PRF and EPF in the posterior region of the kidney supports the long‐recognized possibility that the RF is composed of multiple layers rather than a single continuous sheet. This finding aligns with those described by Marks et al. [9], who reported that the PRF comprises inner and outer layers; the inner layer was continuous with the ARF, whereas the outer layer merged with the LCF. However, the study by Marks et al. [9], involved mechanical dissection, which may have compromised the preservation of interfascial relationships and continuity of adipose tissue compartments. In contrast, we examined intact cadaveric specimens without prior dissection, enabling histological visualization of a clearly delineated and continuous TAC enclosed between the PRF and EPF. Furthermore, according to recent histological studies, the Gerota fascia exhibits a “sandwich” structure, consisting of a central dense collagenous core flanked by looser connective tissue layers [19]. If such a layered configuration applies to the ARF and PRF, the EPF may share a similar architecture. Accordingly, we observed the EPF as an independent fascial structure, supporting the notion that compartmental boundaries in the retroperitoneal space are formed through a gradual and continuous transition from adipose tissue to loose fibrous connective tissue and finally to dense fibrous connective tissue. In retroperitoneal laparoscopic radical nephrectomy, urologists often refer to the incised membrane as the LCF; in this study, this surgically incised membrane corresponds to the EPF, although it is not identical to the classical anatomical definition of the LCF, which refers to the lateral fusion of the ARF and PRF [4].

**FIGURE 5:**
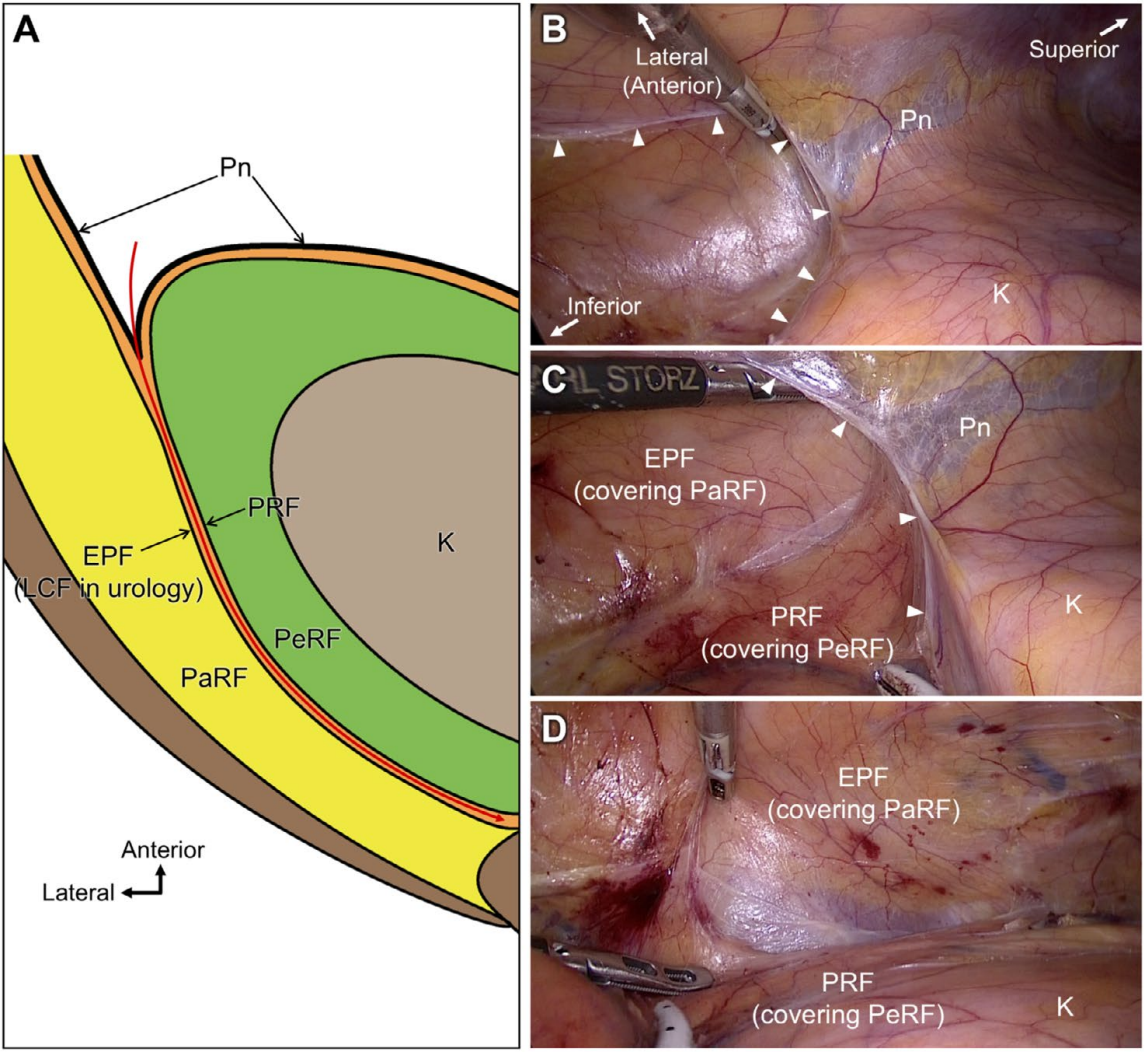
Intraoperative observations during laparoscopic right nephrectomy. Intraoperative photographs of laparoscopic radical nephrectomy performed for a T1a right cystic renal cell carcinoma in a 49‐year‐old female patient undergoing hemodialysis. (A) Overview of the operative field from a transperitoneal approach, illustrating a horizontal cross‐sectional perspective of the right retroperitoneal region. The red line indicates the dissection plane. (B) Identification and incision of the peritoneal reflection at the lateral aspect of the right kidney (white arrowheads). Arrowheads indicate the margins of the incised peritoneum. (C) After dissection between the kidney and the lateral abdominal wall, two distinct fascial layers became clearly visible: Laterally, the extraperitoneal fascia (EPF) enclosing the pararenal fat (PaRF); and medially, the posterior renal fascia (PRF) surrounding the perirenal fat (PeRF). (D) A dissectible plane—corresponding to the TAC—extended posteriorly between these fascial layers, enabling mobilization of the kidney and PeRF while preserving the PRF. These intraoperative findings correspond closely to the compartmental anatomy elucidated in this study. K, kidney; Pn, peritoneum.

Our findings offer valuable insights into clarifying anatomical landmarks during laparoscopic and robot‐assisted urological procedures. Traditionally, surgical dissection around the kidney relies on the anterior and posterior layers of the PRF as key landmarks. However, in actual surgical fields, the fascial structures on the lateral and posterior sides of the kidney are often not perceived as a single continuous layer but rather as multilayered tissue planes. This study identified a TAC between PaRF and PeRF, bounded by the EPF posterolaterally and PRF anteromedially. The EPF, which extends to the posterior aspect of the kidney, is sometimes referred to as the LCF in urology [18]. Our observations are consistent with the multilaminar fascial appearance frequently encountered intraoperatively during laparoscopic radical nephrectomy (Figure 5). By contrast, during retroperitoneal approaches, the EPF and PRF must be incised to access the PeRF from the PaRF side, highlighting the importance of structural understanding in such approaches (Figure SS1, Video.1).

Notably, in patients with reduced visceral fat, the TAC between the EPF and PRF may appear diminished, and the two fascial layers may be closely apposed, mimicking a single fascial membrane. In our observations, the TAC gradually thinned toward the medial posterior side of the kidney and eventually lost its adipose content, at which point the EPF and PRF merged. The exact site of this convergence was inconsistent and varied among the examined cadavers. In the representative operation, EPF and PRF were particularly difficult to separate around the anterior surface of the psoas muscle (PM) (Figure 5). The histological “convergence point” should not be equated with the surgical point of inseparability, and future studies are warranted to examine these two phenomena separately. Thus, the number of fascial layers per se is not a critical issue; rather, recognizing the existence of the TAC as a distinct space is key to accurately identifying the fascial boundaries that define it. These fascial structures should not be understood as primary anatomical entities but as boundary‐forming elements of the adipose compartments they enclose. Clear identification of the interposed adipose compartment and its surrounding fascial layers provides a practical framework for safe and anatomically precise dissection during minimally invasive renal surgery.

This study has some limitations. First, the analysis was limited to the posterior and lateral regions of the kidney. Additionally, this study did not specifically examine the detailed histological relationships between the EPF and the fasciae of the quadratus lumborum and PM, which should be clarified in future investigations. Second, as no systematic assessment of individual variability was conducted. The cadavers used in this study were elderly men. However, the representative surgical case presented in Figure 5 was a female patient, and based on the authors' operative experience, the intraoperative appearance of the adipose compartments and their surrounding fasciae is consistent in men and women. Third, the study was based on static observations of formalin‐fixed cadavers. Accordingly, further validation of the anatomical findings in this study through correlation with intraoperative observations and radiological imaging is warranted.

In conclusion, our macroscopic and histological analysis findings demonstrated that the EPF and PRF exist as two distinct layers of dense fibrous connective tissue with a thin interposed adipose compartment between them. This adipose tissue compartment, which has not been fully recognized in previous anatomical literature, exhibits a distribution distinct from that of PaRF and PeRF. This structure was recognized as TAC. These results suggest that understanding the retroperitoneal anatomy in terms of adipose compartment configuration, rather than relying solely on the classical concept of Gerota's fascia as a single continuous membrane, may offer a more intuitive and practical framework for anatomical orientation.

## Author Contributions

**Atsuhiko Ochi:** conceptualization, writing – review and editing, writing – original draft, investigation, data curation, formal analysis. **Satoru Muro:** conceptualization, writing – original draft, writing – review and editing, investigation, validation, methodology, software, formal analysis, data curation, supervision, visualization. **Sho Mitsumaru:** investigation, validation, visualization, writing – review and editing. **Akimoto Nimura:** visualization, writing – review and editing. **Keiichi Akita:** visualization, writing – review and editing, resources, supervision.

## Funding

Approval of the Research Protocol by an Institutional Review Board: The Human Subjects Research Ethics Review Committee of the Institute of Science, Tokyo, Japan (approval number: M2019‐075). All procedures were conducted in accordance with relevant guidelines and regulations.

## Consent

All donors voluntarily agreed that their remains would be used for educational and research purposes before their death. Written informed consent was obtained from the bereaved families, and no objections were raised.

## Conflicts of Interest

The authors declare no conflicts of interest.

## Supporting information


**Figure S1:** Intraoperative observations during left retroperitoneal laparoscopic living‐donor nephrectomy. Intraoperative photographs of a left retroperitoneal laparoscopic living‐donor nephrectomy in a 57‐year‐old male. (A) Overview of the operative field via a retroperitoneal approach, showing a horizontal cross‐sectional view of the left retroperitoneal region. Red line indicates the dissection plane. (B) The pararenal fat (PaRF) is dissected from the extraperitoneal fascia (EPF, also known as the lateroconal fascia in urology). (C) The EPF is incised, revealing a thin adipose compartment (TAC). Red dashed line indicates the incision margin of the EPF. (D) The posterior renal fascia (PRF) becomes visible within the TAC. Red dashed line indicates the incision margin of the EPF. (E) The PRF is incised, exposing the perirenal fat (PeRF). The PeRF is dissected within the PRF. Red dashed line indicates the incision margin of the EPF, and blue dashed line indicates the incision margin of the PRF. K, kidney; Pn, peritoneum; PM; psoas muscle.

**Video 1** Intraoperative video of a left retroperitoneal laparoscopic living‐donor nephrectomy in a 74‐year‐old male. At the beginning of the video, the pararenal fat (PaRF) is dissected from the extraperitoneal fascia (EPF), also known as the lateroconal fascia in urology. At approximately 0:20, the EPF is bluntly incised, revealing a thin adipose compartment (TAC). At around 0:30, the posterior renal fascia (PRF) is retracted upward, partially exposing the psoas muscle (PM). At approximately 1:15, the PRF is electrically incised using scissors. From this point onward, dissection continues within the PRF, along the loose connective tissue on the outer surface of the perirenal fat (PeRF). The PM surface remains covered by the PRF and EPF, preventing exposure of the muscle fibers.

## Data Availability

Data supporting the findings of this study are available from the corresponding author upon request.
