# Supplementary figures and images for: Anatomy of Adipose Compartments and Fascial Structures in the Posterolateral Region of the Kidney With Special Focus on the Thin Adipose Compartment
Source: Int J Urol. 2025 Nov 27;33(2):e70303. doi: 10.1111/iju.70303 (PMC12854919; doi:10.1111/iju.70303)

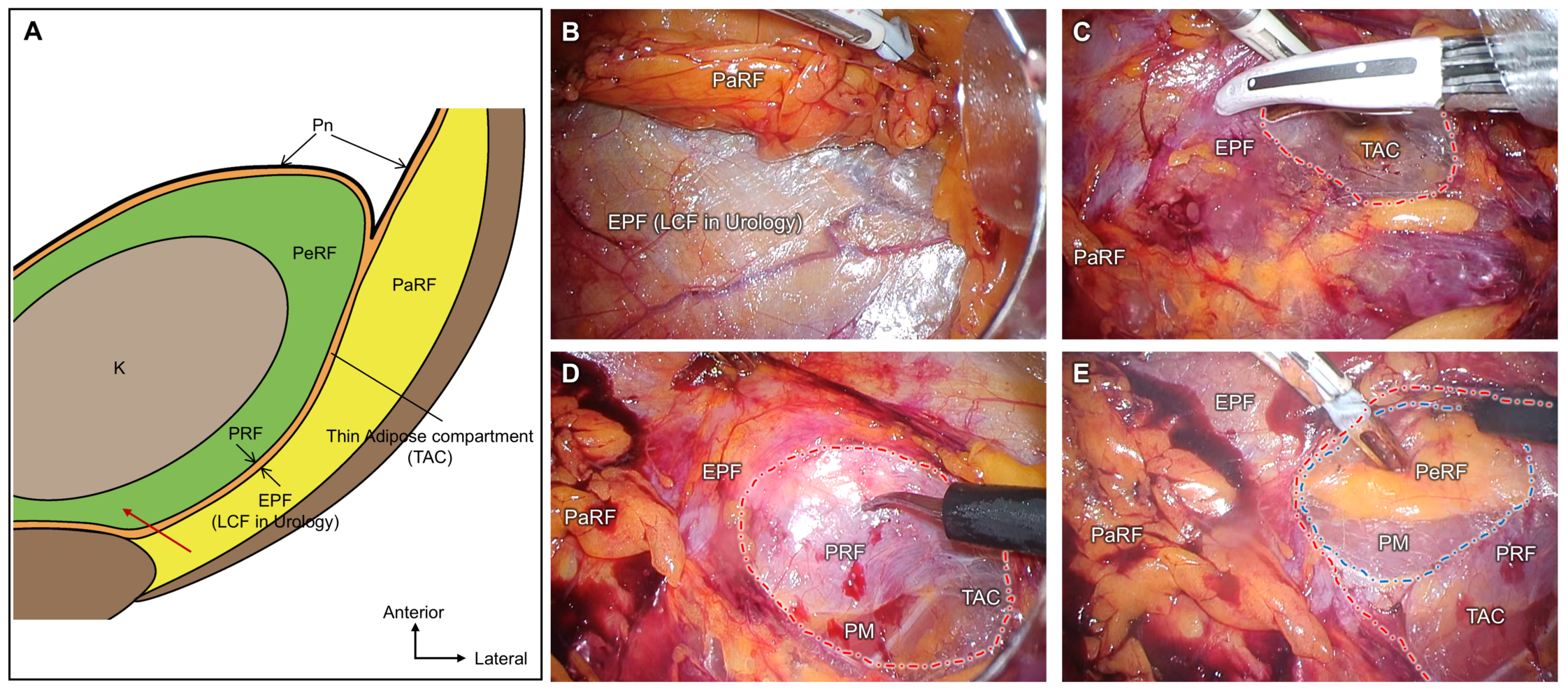

Supplement: Supplementary file 1 — Figure S1: Intraoperative observations during left retroperitoneal laparoscopic living‐donor nephrectomy. Intraoperative photographs of a left retroperitoneal laparoscopic living‐donor nephrectomy in a 57‐year‐old male. (A) Overview of the operative field via a retroperitoneal approach, showing a horizontal cross‐sectional view of the left retroperitoneal region. Red line indicates the dissection plane. (B) The pararenal fat (PaRF) is dissected from the extraperitoneal fascia (EPF, also known as the lateroconal fascia in urology). (C) The EPF is incised, revealing a thin adipose compartment (TAC). Red dashed line indicates the incision margin of the EPF. (D) The posterior renal fascia (PRF) becomes visible within the TAC. Red dashed line indicates the incision margin of the EPF. (E) The PRF is incised, exposing the perirenal fat (PeRF). The PeRF is dissected within the PRF. Red dashed line indicates the incision margin of the EPF, and blue dashed line indicates the incision margin of the PRF. K, kidney; Pn, peritoneum; PM; psoas muscle. [file IJU-33-0-s001.tif]
